# Practical Medicine

**Published:** 1878-02

**Authors:** 


### Practical Medicine.

Atrophy of the Testicles after Mumps.—M. Lereboullet, at a meeting of the Hospital Society (*Bulletin Gen. de Thérap.*, September, 1877, *Phil. Med. Times*), presented a soldier, aged 22 years, who, four months previously, had been attacked by mumps. At that time he had every appearance of virility, but four days afterwards double orchitis occurred, under the influence of which the testicles swelled until they were each the size of a fist. The organs subsequently became atrophied until they were reduced to the volume of an almond; at the same time there was evident a considerable development of the mammary glands. The beard also was arrested in its growth, and the patient had a perfectly smooth chin as a result of this physiological process of depilation.

Gout Treated by the Cold Water Douche.—Dr. C. G. Rothe (*Memorabilien*, 1877, No. 11). Four cases are reported to show the beneficial effect of cold water upon the gouty inflammation. All the writer claims is that this treatment quickly allayed the pain and decidedly shortened the attacks.

Here is one of the cases: A physician, aged 50 years, was, in February, 1876, suddenly seized at night by violent pain in the joint of his left great toe. At first, he attributed it to an injury he had received a few days previously; but the utter inefficiency of the ordinary antiphlogistics, and the hard exudations about the joint, soon convinced him of the gouty nature of his trouble. The attack lasted three weeks, and the toe remained sensitive and stiff several months.

In January, 1877, he had a second attack in the right toe. The joint was swollen, immovable, would not bear the slightest touch, the skin purple and glistening.

On the advice of Dr. R., the patient tried the cold water douche. He put his foot under the cold water faucet and allowed the jet to play on the painful joint during three minutes. The pain subsided instantly, and an agreeable sensation of warmth crept over the foot, which was rubbed dry after the douche. Two hours later, when the pain returned, though with less violence than before, the douche was re-applied, and repeated every two or three hours during that day. The patient passed a very good night. The next morning, the swelling and redness about the toe was gone, and the joint was a little sensitive only on strong flexion. That day, the douche was used four or five times; but since, the patient has attended to his practice and been entirely free of gout.

CATARRHAL JAUNDICE TREATED BY ENEMATA OF COLD WATER. (*Bulletin Gén. de Thérap.*, September, 1877, *Phil. Med. Times.*) As soon as the diagnosis of catarrhal jaundice is made, Dr. Krull, of Mecklenburg, commences injecting two to four pints of water into the rectum by means of irrigation. The water should be about 59° F. in temperature, and the operation should be practised once in twenty-four hours. When the enema is repeated, the temperature should be raised a few degrees, because the intenstine does not bear well, repeated contact with water of the same degree. The patient should be instructed to retain the fluid in the bowel as long as it is possible for him to do so. The notes of eleven cases are given, showing that, after a few injections in the manner described, the stools became colored with bile, the tenderness in the hepatic region, the malaise and headache disapeared, and the anorexia was notably decreased. A cure usually resulted after the administration of seven cold-water injections, and is supposed to be due to the stimulation of the peristaltic action of the intestines and to the excitation of the biliary secretion, which, by its increased quantity in the passages, overcomes the obstacle to its free escape.

A Peculiar Case of Variola is reported by Dr. Kersch in his *Observations During the Small-pox Epidemic in Prague*, 1876 (Memorabilien 1877, No. 11). On October 22, 1876, he was summoned to attend Mr. C. F., 28 years of age, a robust, broad-shouldered man, who had never been sick before. On the previous morning he was apparently well, and attended to his business of civil engineer; but before dinner he suddenly felt sick, and fainting, fell from his chair. The most prominent subjective symptoms were: headache, stupor, great restlessness, violent thirst. The objective symptons were: pulse, 126; temperature, 39.5°; face flushed, lips dry and purple, tongue heavily coated and dry, internal organs normal except the spleen, which was slightly enlarged; the urine highly colored and saturated with the salts of uric acid. The first night the patient was very restless, the second night he became delirious. At 10 o'clock in the evening of Oct. 24, the doctor was sent for, because the patient had become so wild that he could not be kept in bed. It was necessary to put a jacket on and to tie him to the bed. He drank eagerly if water was brought to his lips; still his lips and tongue were dry like parchment, brown and furred. Toward morning he became quieter, and slept a few hours. The next day and night he passed in the same wild delirium as before; but on the morning of Oct. 26 his mind was very much clearer; pulse 120, temperature 38.3°. The most scrupulous search for any cutaneous efflorescence resulted in the discovery of one single minute papule, surrounded by a red halo. While this was gradually growing into a regular variola pustule the fever abated, and when it was completely developed, the fever and all the constitutional symptoms disappeared.

